# Retrospective Case-Control Study of Risk Factors for Carbapenem-Resistant *Klebsiella pneumoniae* Infection in Children in China

**DOI:** 10.3390/pathogens13121106

**Published:** 2024-12-14

**Authors:** Caizhen Wang, Lijie Feng, Ruomu Chen, Yuan Chen

**Affiliations:** 1 Pediatric Intensive Care Unit, The Second Hospital of Hebei Medical University, Shijiazhuang 050050, China; paediatrician2021@163.com (C.W.); fenglijie_2015@163.com (L.F.); 2 College of Basic Medical Sciences, Hebei Medical University, Shijiazhuang 050017, China; ruomuch@163.com

**Keywords:** carbapenem-resistant *Klebsiella pneumoniae*, pediatric infections, risk factors, clinical outcomes, mortality

## Abstract

This study aims to investigate the risk factors for infection and mortality associated with carbapenem-resistant *Klebsiella pneumoniae* (CRKP) in hospitalized children, with the goal of providing valuable insights for the prevention and treatment of these bacterial infections. A retrospective case-control study was conducted, including 153 cases of carbapenem-sensitive *K. pneumoniae* infection in children and 49 cases of CRKP infection. Among the CRKP cases, 40 children survived and nine died. Logistic regression analysis was used to screen the risk factors for CRKP infection in children, establish a predictive model, and analyze the factors associated with mortality in CRKP-infected children. The results of the multivariate regression analysis showed that hematopoietic malignancies (OR = 28.272, 95% CI: 2.430–328.889), respiratory tract infections (OR = 0.173, 95% CI: 0.047–0.641), mechanical ventilation (OR = 3.002, 95% CI: 1.117–8.071), number of antibiotic agents (OR = 1.491, 95% CI: 1.177–1.889), WBC (OR = 0.849, 95% CI: 0.779–0.926), and neutrophil count (OR = 0.779, 95% CI: 0.677–0.896) were identified as significant factors associated with CRKP infection in children. Specifically, CRKP-infected children with a history of multiple hospitalizations within the past three months, blood stream infections, and decreased WBC and lymphocyte counts should be monitored closely due to poor prognosis. Underlying hematopoietic malignancies in children, non-respiratory tract infections, mechanical ventilation after admission, and use of multiple antibiotics without significant increase in white blood cell and neutrophil counts are major factors influencing CRKP infection. Particularly, CRKP-infected children with blood stream infections and no significant increase in neutrophil count should be closely monitored for potential severity of illness.

## 1. Introduction

In recent years, the detection rate of carbapenem-resistant Enterobacterales bacteria has been increasing annually due to the widespread and indiscriminate use of carbapenem antibiotics [1]. According to surveillance data from the China Antimicrobial Resistance Surveillance System, the proportion of carbapenem-resistant *Klebsiella pneumoniae* (CRKP) isolates within the Enterobacteriaceae family rose rapidly from 3% in 2005 to 25.4% in 2019, representing an eightfold increase [2]. The detection rate of CRKP in children has also been steadily rising, increasing from 5.6% in 2014 to 9.6% in 2018 [3]. *Klebsiella pneumoniae* (*K. pneumoniae*) is a Gram-negative bacterium that is commonly responsible for a variety of infections, including bloodstream infections, pneumonia, urinary tract infections, and intra-abdominal infections [4]. It is a major cause of nosocomial infections, particularly in vulnerable patient populations such as children, the elderly, and those with compromised immune systems [5]. CRKP is known for its resistance to multiple antibiotics, which contributes to increased treatment failure rates and mortality rates ranging from 20% to 70%. While the virulence of CRKP is significant, the primary concern is its high level of antibiotic resistance [6,7,8,9]. The increasing prevalence of antimicrobial resistance (AMR) globally poses a significant public health threat [10]. AMR complicates the treatment of infectious diseases, limits the effectiveness of standard antibiotics, and results in higher healthcare costs, longer hospital stays, and greater mortality [11]. *K. pneumoniae*, particularly in its resistant form, has become a key pathogen in the AMR crisis [12].

In children, risk factors for CRKP infection and mortality have been increasingly studied, though there remain significant differences between the pediatric and adult populations [13]. Risk factors for CRKP infection in children often include prolonged hospital stays, mechanical ventilation, central venous catheterization, and immunocompromised conditions such as hematologic malignancies [8]. Recent studies from China and other countries have highlighted that children with underlying chronic conditions, such as cancer or congenital immunodeficiencies, are particularly susceptible to CRKP infections [14]. In addition, the widespread use of carbapenem antibiotics in pediatric intensive care units (ICUs) has been associated with a higher incidence of CRKP, underscoring the importance of antibiotic stewardship in managing these infections [15]. However, there are still gaps in the literature regarding the specific risk factors for CRKP mortality in children. Several studies have suggested that comorbidities such as sepsis, respiratory failure, and multi-organ dysfunction are major contributors to CRKP-related mortality [16]. There is also ongoing debate over the optimal timing and selection of antibiotics for pediatric CRKP infections, with some advocating for earlier broad-spectrum antibiotic administration, while others emphasize the need for tailored treatment based on culture and susceptibility results [17,18]. What is known from existing research is that early identification of risk factors, including mechanical ventilation, prolonged antibiotic use, and underlying immunocompromising conditions, can help guide clinical management and improve outcomes [19]. However, controversy persists regarding the role of specific microbiological characteristics, such as the presence of certain virulence factors in CRKP strains in influencing clinical outcomes. Therefore, a clearer understanding of these risk factors, as well as a more standardized approach to treatment, is crucial for improving the management of pediatric CRKP infections.

While much of the literature focuses on CRKP infections in adult populations, the clinical manifestations, risk factors, and outcomes may differ significantly in children due to their distinct physiological and immunological characteristics. Unlike adults, children’s immune systems are still developing, making them more vulnerable to severe infections and less capable of mounting effective immune responses [20]. Additionally, younger children may have immature organ systems, leading to different pharmacokinetics and pharmacodynamics when compared to adults, further complicating treatment strategies. Carbapenems, such as imipenem, meropenem, and ertapenem, are essential antibiotics used to treat severe infections caused by multi-drug-resistant pathogens, including *K. pneumoniae* [21]. Resistance to carbapenems is particularly alarming because these drugs are often considered the last line of defense against resistant Gram-negative bacteria [22]. The emergence of CRKP, therefore, represents a serious threat to public health and underscores the need for novel therapeutic strategies. The existing body of research on pediatric CRKP infections is relatively limited, highlighting the need for more comprehensive studies to better understand the clinical characteristics, prognosis, and specific risk factors associated with poor outcomes in children. As CRKP infections in children often involve severe complications such as sepsis, respiratory failure, and multi-organ dysfunction, the disease burden and therapeutic challenges in this age group are significant. Therefore, this study adopts a case-control research design to conduct a retrospective investigation of CRKP infections in children, analyzing the risk factors associated with infection and mortality. The aim is to provide a basis for the prevention and control of CRKP infections in children. Through an in-depth analysis of pediatric CRKP infections, this research aims to offer clinicians more effective treatment strategies and preventive measures to reduce disease progression and mortality rates among pediatric patients, addressing the significant challenges faced in pediatric healthcare.

## 2. Materials and Methods

### 2.1. Study Design

This study employed a case-control research method to retrospectively collect basic clinical information on pediatric patients infected with carbapenem-sensitive *K. pneumoniae* (CSKP) and CRKP admitted to the Second Hospital of Hebei Medical University, a tertiary teaching hospital with approximately 2816 inpatient beds, serving as a regional referral center for pediatric care, from January 2018 to December 2023. The study compared the similarities and differences in factors associated with CRKP and CSKP infections in children, analyzed the risk factors for CRKP infection, established a predictive model for CRKP infection, and concurrently examined the risk factors for mortality among CRKP-infected children. This study was approved by the Ethics Committee of the Second Hospital of Hebei Medical University (Approval No. 2024-R253). Informed consent was obtained from all subjects involved in the study.

### 2.2. Inclusion and Exclusion Criteria

Inclusion criteria: (1) Age between 28 days and 14 years old; (2) Diagnosis of *K. pneumoniae* infection, categorized as either CSKP or CRKP based on susceptibility testing results; (3) Complete medical records available.

Exclusion criteria: (1) Patients not diagnosed with *K. pneumoniae* infection; (2) Pediatric patients with repeated *K. pneumoniae* infections were only included based on their first positive result in this study.

### 2.3. Data Collection

Clinical data from the three months preceding the diagnosis of *K. pneumoniae* infection were retrieved from the hospital’s electronic medical record management system and organized using Excel (2021) spreadsheets. The collected clinical information included the following: (1) Demographics: age and gender; (2) Underlying diseases diagnosed before infection: hematopoietic malignancies, congenital malformations, neurological disorders, multiple organ dysfunction syndrome (MODS), etc.; (3) Sites of infection: respiratory tract infection, bloodstream infection, urinary tract infection, etc. Respiratory tract infection was defined as an infection characterized by clinical signs such as coughing, wheezing, and abnormal lung sounds, confirmed by a positive sputum culture or bronchoalveolar lavage culture for *K. pneumoniae*. Bloodstream infection was defined as the presence of *K. pneumoniae* in at least one positive blood culture from a patient with clinical symptoms of sepsis. Urinary tract infection was defined as *K. pneumoniae* identified in a urine culture from a symptomatic patient, such as one presenting with fever, dysuria, or flank pain; (4) Relevant treatments before infection: Mechanical ventilation was defined as the use of invasive or non-invasive respiratory support for more than 24 h. Central venous catheterization was defined as the insertion of a catheter into a central vein for more than 48 h. Glucocorticoid therapy was defined as the administration of systemic glucocorticoids for more than 5 days; (5) Positive bacterial culture results and concurrent laboratory findings: white blood cell count (WBC), absolute neutrophil count, absolute lymphocyte count, etc.; (6) Hospitalization-related information: length of hospital stay was calculated in days from the date of admission to the date of discharge or death. Mortality rate was defined as the percentage of patients who died within 7 or 28 days following CRKP infection; (7) Antibiotic use prior to infection: clinical data on antibiotic use over the three months preceding the diagnosis of *K. pneumoniae* infection were collected from hospital records.

### 2.4. Carbapenem Resistance Detection

The bacterial identification was performed using the Vitek 2 Compact automated microbiological analysis system or the Vitek MS mass spectrometry system with corresponding reagents, both provided by bioMérieux (Marcy-l’Étoile, France). Antimicrobial susceptibility testing (AST) was conducted using the Vitek 2 Compact automated microbiological analysis system and its associated reagents. Quality control strains included *Escherichia coli* ATCC 25922 and *Pseudomonas aeruginosa* ATCC 27853. The interpretation of *K. pneumoniae* susceptibility testing followed the standards established by the Clinical and Laboratory Standards Institute (CLSI) [23], defining carbapenem-resistant *K. pneumoniae* (CRKP) as isolates meeting any of the following minimum inhibitory concentration (MIC) criteria: imipenem MIC ≥ 4 μg/mL, meropenem MIC ≥ 4 μg/mL, or ertapenem MIC ≥ 2 μg/mL.

### 2.5. Sample Size and Frequency

A total of 153 pediatric patients with CSKP and 49 patients with CRKP were included in this study. For each patient, clinical data were collected at the time of diagnosis of *K. pneumoniae* infection, and follow-up data were recorded until discharge or death. Data collection was performed retrospectively, with samples being reviewed from the electronic medical record management system for the specified period. Samples were taken and analyzed based on routine clinical practices and diagnostic requirements at the time of admission and throughout the hospital stay. Routine clinical practices included a comprehensive evaluation of the patient’s clinical symptoms, physical examination, and laboratory tests such as urine cultures, blood cultures, and imaging studies. Diagnostic requirements included the use of microbiological culture methods to identify *K. pneumoniae*, along with antibiotic susceptibility testing following the CLSI guidelines to confirm the infection and guide treatment decisions.

### 2.6. Antibiotic Treatment

Patients in this study received various antibiotics based on clinical indications and susceptibility testing results. Commonly administered antibiotics included carbapenems (e.g., imipenem, meropenem), cephalosporins, aminoglycosides, and fluoroquinolones. The choice of antibiotics was determined by the treating physician based on the patient’s condition and the sensitivity profile of the *K. pneumoniae* isolate.

### 2.7. Statistical Analysis

Statistical analysis was performed using SPSS 24 and R4.3.2 software to ensure robust data handling and statistical processing. The analysis aimed to identify and quantify risk factors associated with CRKP infection and mortality, and to establish a predictive model for CRKP infection. Non-normally distributed continuous variables were summarized using median and interquartile range [M (IQR)]. Categorical variables were summarized as counts and percentages (%). These descriptive statistics provided an overview of the demographic and clinical characteristics of the study population. To compare differences between groups (CRKP vs. CSKP infections), non-normally distributed continuous variables were analyzed using the Mann–Whitney U test. Categorical variables were compared using the chi-squared test. These comparisons helped to identify initial associations and differences between CRKP and CSKP infections. Bivariate analysis was conducted to identify potential risk factors for CRKP infection and mortality. Variables with a *p*-value < 0.05 in bivariate analysis were considered for inclusion in the multivariate model. Multivariate logistic regression models were used to identify independent risk factors for CRKP infection and mortality, adjusting for potential confounders. The backward stepwise selection method was employed to refine the model by sequentially removing non-significant variables, ensuring that only the most relevant predictors were included. This approach helped to determine the independent effects of each variable on the outcomes. Significant factors from the multivariate logistic regression were used to develop a predictive model for CRKP infection. The performance of the predictive model was assessed using ROC curves, with the area under the ROC curve (AUC) providing a measure of the model’s discriminatory ability. All statistical tests were two-tailed, and a *p*-value < 0.05 was considered statistically significant. This significance level was chosen to balance the risk of Type I and Type II errors in identifying meaningful associations.

## 3. Results

### 3.1. Clinical Characteristics of the Two Groups of Patients

A total of 153 children were infected with CSKP, and 49 children were infected with CRKP. Significant differences were observed between the two groups of patients in terms of hematopoietic malignancies, respiratory tract infection, bloodstream infection, mechanical ventilation, central venous catheterization, number of antibiotic agents, white blood cell count (WBC), neutrophil count, lymphocyte count, length of stay, 7-day mortality, 28-day mortality, and hospital mortality (*p* < 0.05). There were no statistically significant differences between the two groups in terms of age, sex, congenital malformation, and neurological disorders (*p* > 0.05) (Table 1).

### 3.2. Antibiotic Use Prior to the Release of Susceptibility Results

The median number of antibiotic types used prior to the release of the susceptibility results was four in children infected with CRKP, which was significantly higher than the median of two in children infected with CSKP. The results indicated a statistically significant difference in the use of carbapenems, with 13.7% of CSKP patients and 57.1% of CRKP patients receiving carbapenem antibiotics (*p* < 0.001). The frequency of second-generation cephalosporin use was 63.3% in the CRKP group, higher than the 47.1% in the CSKP group, with a statistically significant difference (*p* = 0.048). The proportion of CRKP patients using third-generation cephalosporins (77.6%) was higher than that of CSKP patients (62.7%) (*p* = 0.004). Significant differences were also observed in the use of glycopeptides and oxazolidinones, with usage rates of 24.5% and 26.5%, respectively, in the CRKP group, compared to 4.6% and 2.0% in the CSKP group (*p* < 0.001). No significant differences were found in the use of other types of antibiotics between the two groups (Table 2).

### 3.3. Analysis of Risk Factors for CRKP Infection in Children

Single-factor and multi-factor logistic regression analyses were conducted on hematopoietic malignancies, respiratory tract infection, bloodstream infection, mechanical ventilation, central venous catheterization, number of antibiotic agents, white blood cell count (WBC), neutrophil count, and lymphocyte count. The results of the multivariate regression analysis showed that hematopoietic malignancies (OR = 28.272, 95% CI: 2.430–328.889), respiratory tract infection (OR = 0.173, 95% CI: 0.047–0.641), mechanical ventilation (OR = 3.002, 95% CI: 1.117–8.071), number of antibiotic agents (OR = 1.491, 95% CI: 1.177–1.889), WBC (OR = 0.849, 95% CI: 0.779–0.926), and neutrophil count (OR = 0.779, 95% CI: 0.677–0.896) were identified as significant factors associated with CRKP infection in children (Table 3).

### 3.4. Establishment of Risk Model for Pediatric CRKP Infection

**Figure 1:**
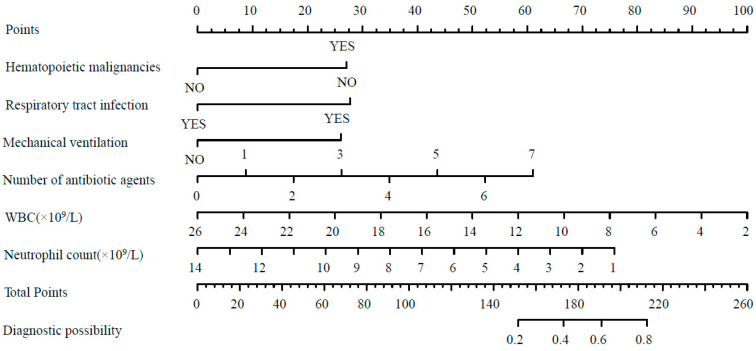
Predictive risk model for identifying CRKP infection in pediatric patients.

**Figure 2:**
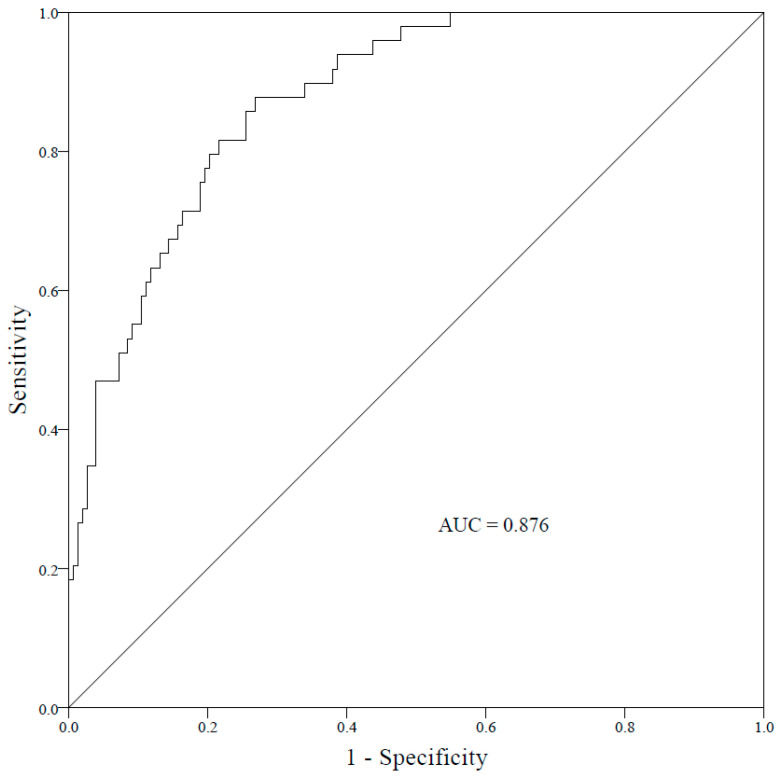
Receiver operating characteristic (ROC) curve for the risk prediction model of pediatric CRKP infection.

Based on the six factors selected through multi-factor logistic regression, a forest plot model was constructed (Figure 1). By summing the scores of the respective predictive factors, the total score corresponds to the probability of pediatric CRKP infection occurrence. The forest plot prediction model for CRKP infection risk showed an ROC curve result with an AUC of 0.876 (95% CI = 0.827–0.926), indicating good predictive accuracy (Figure 2).

### 3.5. Analysis of Factors Associated with Mortality in CRKP-Infected Children

Among the 49 children infected with CRKP, there were nine non-survivors and 40 survivors. A comparison between the two groups revealed that non-survivors had a higher proportion of bloodstream infections, decreased white blood cell (WBC) and lymphocyte counts, and a significantly higher number of hospitalizations in the previous 3 months (*p* < 0.05) (Table 4).

## 4. Discussion

*K. pneumoniae* is prevalent globally with a rising incidence, garnering significant attention from clinicians. This upward trend is driven by two primary factors: increasing detection rates of drug-resistant strains posing greater challenges in antibiotic selection for clinicians, and a notably high mortality rate associated with CRKP infections [24,25]. However, studies on CRKP infections in children remain relatively scarce. Therefore, this study aimed to collect the clinical data of pediatric patients with *K. pneumoniae* infection and conduct a retrospective analysis to explore their clinical characteristics and mortality risk factors. The study’s significance is highlighted by its contribution to early empirical treatment recommendations, which is particularly crucial in pediatric populations where CRKP data are limited. By providing insights into high-risk profiles, such as those with hematologic malignancies or recent mechanical ventilation, this research supports more informed decision-making in initial antibiotic therapy.

In terms of demographics, no significant differences were found between patients with CRKP and CSKP infections. However, other studies have shown that CRKP infections tend to affect younger children more frequently than CSKP infections, likely due to the more severe underlying conditions associated with CRKP, such as immunocompromised states and prolonged hospitalizations [26,27]. This difference may be attributed to the diverse infection sites covered in this study, including respiratory tract, bloodstream, and urinary tract infections. Although urinary tract infections (UTIs) were considered when characterizing the cohort, they were not included in the multivariate analysis due to the limited sample size and their relatively low occurrence rate. Additionally, considering the substantial differences in underlying conditions, prior treatments, and diagnostic results between neonates and older children, this study excluded neonatal patients. Regarding infection sites, both CSKP and CRKP patients exhibited the highest proportion of respiratory tract infections. However, the significantly higher proportion of bloodstream infections in CRKP patients aligns with findings from other studies that highlight the increased severity and mortality risk associated with bloodstream infections in this group [28,29]. Observations regarding underlying conditions and pre-infection treatments revealed a higher proportion of hematologic malignancies among CRKP-infected patients. This may be due to the need for chemotherapy, steroids, and immunosuppressants in patients with hematologic malignancies, which can compromise immune function and increase the risk of bloodstream infections [30,31]. This is consistent with previous studies that emphasized the vulnerability of immunocompromised children to CRKP due to their weakened immune responses, particularly following treatments such as chemotherapy or long-term use of immunosuppressive drugs [32]. The specific types of antibiotics these patients received included broad-spectrum agents such as carbapenems, cephalosporins, and aminoglycosides [33]. Administration was often empirical, as susceptibility results were not always immediately available, highlighting the challenge of aligning treatment with timely laboratory data. Moreover, the use of multiple types of antibiotics by these patients in recent times may contribute to the emergence of resistant strains, as discussed by several studies emphasizing the role of antibiotic misuse in AMR development [34].

In line with previous studies, mechanical ventilation and the use of central venous catheters are key risk factors for CRKP infections in pediatric populations, particularly due to their role in facilitating bacterial entry and evading host immune responses [20,35]. This phenomenon may be attributed to compromised natural barriers in the body, as *K. pneumoniae* has highly adhesive fimbriae that can easily attach to medical device surfaces, providing a pathway for entry into the human body [36,37]. Additionally, the increased hospitalizations observed in the CRKP group may be associated with the higher prevalence of mechanical ventilation and invasive procedures. Studies indicate a significant increase in the risk of *K. pneumoniae*-related ventilator-associated pneumonia after endotracheal intubation exceeding 48 h, likely due to disruption of respiratory tract clearance mechanisms. During infection, a notable increase in white blood cells and neutrophils may be in response to underlying conditions [38]. However, a significant decrease in these cell counts may lead to poor infection control, thereby promoting the emergence of resistant strains. Children, being a special demographic with immature immune systems, exhibit lower resistance to external pathogens and are therefore at increased risk of exposure in hospital environments [8]. Based on these findings, we recommend that children with underlying hematologic malignancies, especially those who have undergone mechanical ventilation or central venous catheterization, and whose blood test results show no significant increase in white blood cells or neutrophils, be categorized as a high-risk group for CRKP colonization. Early proactive screening and appropriate interventions should be implemented to prevent or promptly address CRKP infections.

In our study of CRKP-related mortality factors, we observed a significant increase in the proportion of bloodstream infections among deceased patients, accompanied by notable decreases in white blood cell and lymphocyte counts. Additionally, these patients had multiple hospitalizations within the previous 3 months. This phenomenon may be attributed to lower immune function and more severe illness among patients with bloodstream infections, consistent with findings from other studies [24,39]. Several studies have similarly identified low white blood cell counts and multiple hospitalizations as independent risk factors for poor outcomes in CRKP infections, with bloodstream infections being most commonly associated with mortality [40]. However, we recognize that the significantly higher proportion of bloodstream infections in the CRKP group compared to the CSKP group could lead to biased results when interpreting risk factors. Future studies should address this issue by conducting more rigorous, infection-type-consistent analyses to avoid confounding. Based on these observations, we recommend particular attention to white blood cell counts in CRKP-infected children with recent multiple hospitalizations and bloodstream infections. If the white blood cell count shows no significant increase, prompt treatment should be administered. These observations underscore important clinical management strategies for this patient population, aimed at improving recovery rates and prognosis.

Looking forward, strategies to control the spread of CRKP should include strengthening infection prevention and control measures in healthcare settings, such as more rigorous hygiene practices, proper sterilization of medical equipment, and improved antimicrobial stewardship programs. Given the high-risk populations identified in this study, proactive screening of high-risk groups, especially those with underlying conditions or invasive treatments, should be implemented to detect colonization early and prevent the progression to clinical infection. In addition, the development of new antimicrobial agents, including novel carbapenem derivatives and alternative treatments, is urgently needed to combat the growing threat of CRKP. Collaboration between researchers, clinicians, and public health officials is essential to mitigate the public health impact of CRKP and other multidrug-resistant pathogens.

Due to the single-center nature of this study, the generalizability of the conclusions may be limited, and they may not be fully applicable to other healthcare settings. Additionally, as a retrospective study, it was constrained by the quality and completeness of available medical records, which may have affected the comprehensiveness of the data collection. Future multi-center prospective studies are needed to validate these findings in broader clinical contexts. The relatively small sample size, particularly the limited number of CRKP-infected children, further restricted our ability to conduct certain comparative analyses, such as stratified analysis between survivors and non-survivors, which would have provided deeper insights into mortality-related risk factors. These limitations underscore the need for larger, multicenter prospective studies to validate our findings and allow for more robust comparisons.

## 5. Conclusions

In summary, among pediatric patients, those with a history of hematologic malignancies and non-respiratory tract infections, who undergo mechanical ventilation upon admission and receive multiple antimicrobial agents, but do not exhibit significant increases in white blood cell and neutrophil counts, are primarily affected by CRKP infection. The use of multiple antimicrobial agents in these patients may contribute to the development of CRKP infection. For CRKP-infected children with bloodstream infections and no significant increase in neutrophil count, caution should be exercised as this may indicate potential severity of illness.

## Figures and Tables

**Table 1:** Comparison of clinical characteristics between CSKP and CRKP pediatric patients.

Factor	CSKP (*n* = 153)	CRKP (*n* = 49)	Z/χ^2^	*p* Value
Age (months)	28 (13–60.5)	33 (19–79.5)	−1.729	0.084
Sex			0.327	0.567
Male	74 (48.4)	26 (53.1)		
Female	79 (51.6)	23 (46.9)		
Pre-existing condition				
Hematopoietic malignancies	14 (9.2)	20 (40.8)	26.585	<0.001
Congenital malformation	3 (2.0)	3 (6.1)	1.020	0.313
Neurological disorders	16 (10.5)	5 (10.2)	0.003	0.960
MODS	17 (11.1)	8 (16.3)	0.931	0.335
Site of infection				
Respiratory tract infection	129 (84.3)	30 (61.2)	11.808	<0.001
Bloodstream infection	11 (7.2)	11 (22.4)	8.905	0.003
Urinary tract infection	5 (3.3)	3 (6.1)	0.222	0.638
Infection at other sites	8 (5.2)	5 (10.2)	0.811	0.368
Pre-infection related treatment				
Mechanical ventilation	28 (18.3)	25 (51.0)	20.53	<0.001
Central venous catheterization	28 (18.3)	17 (34.7)	5.76	0.016
Number of antibiotic agents	2 (1–3)	4 (2–5.5)	−3.784	<0.001
Glucocorticoid therapy	34 (22.2)	16 (32.7)	2.168	0.141
Relevant examination results at the time of infection				
WBC (×10^9^/L)	12.1 (8.85–15.7)	9.27 (7.19–11.65)	−3.784	<0.001
Neutrophil count (×10^9^/L)	7.02 (4.94–9.48)	5.74 (4.08–7.15)	−3.295	<0.001
lymphocyte count (×10^9^/L)	3.63 (2.42–4.72)	2.79 (2.06–3.69)	−3.444	<0.001
Hospitalization information				
Length of stay (days)	15 (9.5–22)	27 (14–41)	−4.529	<0.001
7-day mortality	1 (0.7)	3 (6.1)	3.248	0.071
28-day mortality	3 (2.0)	9 (18.4)	15.063	<0.001
Hospital mortality	6 (3.9)	9 (18.4)	9.263	0.002

Data are *n* (%) or median (IQR).

**Table 2:** Comparison of antibiotic use prior to the release of susceptibility results.

Antibiotic Categories	CSKP (*n* = 153)	CRKP (*n* = 49)	χ^2^	*p* Value
Carbapenems	21 (13.7)	28 (57.1)	38.078	<0.001
First-generation cephalosporins	8 (5.2)	2 (4.1)	<0.001	1.000
Second-generation cephalosporins	72 (47.1)	31 (63.3)	3.091	0.048
Third-generation cephalosporins	96 (62.7)	38 (77.6)	8.345	0.004
β-lactamase inhibitors	56 (36.6)	23 (46.9)	1.665	0.197
Penicillins	43 (28.1)	15 (30.6)	0.114	0.736
Aminoglycosides	42 (27.5)	12 (24.5)	0.166	0.684
Quinolones	13 (8.5)	6 (12.2)	0.251	0.616
Glycopeptides	7 (4.6)	12 (24.5)	15.015	<0.001
Oxazolidinones	3 (2.0)	13 (26.5)	27.443	<0.001
Tetracyclines	1 (0.7)	3 (6.1)	3.248	0.071

**Table 3:** Bivariate and multivariable analysis of risk factors associated with CRKP infection.

Factor	Bivariate Analyses		Multivariate Analyses	
	OR (95% CI)	*p* Value	OR (95% CI)	*p* Value
Hematopoietic malignancies	6.847 (3.103–15.111)	<0.001	28.272 (2.430–328.889)	0.008
Respiratory tract infection	0.294 (0.143–0.604)	0.001	0.173 (0.047–0.641)	0.009
Bloodstream infection	3.737 (1.505–9.276)	0.004	/	/
Mechanical ventilation	4.650 (2.323–9.309)	<0.001	3.002 (1.117–8.071)	0.029
Central venous catheterization	2.372 (1.158–4.857)	0.018	/	/
Number of antibiotic agents	1.442 (1.211–1.718)	<0.001	1.491 (1.177–1.889)	0.001
WBC (×10^9^/L)	0.849 (0.779–0.926)	<0.001	0.800 (0.704–0.908)	0.001
Neutrophil count (×10^9^/L)	0.779 (0.677–0.896)	<0.001	0.751 (0.623–0.905)	0.003
Lymphocyte count (×10^9^/L)	0.635 (0.489–0.824)	0.001	/	/

**Table 4:** Clinical characteristics comparison between surviving and non-surviving pediatric patients with CRKP infection.

Factor	Survival (*n* = 40)	Non-Survival (*n* = 9)	Z/χ^2^	*p* Value
Age (months)	35.5 (23.0–80.25)	19 (15–84.5)	−0.828	0.423
Sex			<0.001	1.00
Male	21 (52.5)	5 (55.6)		
Female	19 (47.5)	4 (44.4)		
Pre-existing condition				
Hematopoietic malignancies	15 (37.5)	5 (55.6)	0.385	0.535
Congenital malformation	2 (5.0)	1 (11.1)	<0.001	1.00
Neurological disorders	4 (10.0)	1 (11.1)	<0.001	1.00
MODS	6 (15.0)	2 (22.2)	0.001	0.976
Site of infection				
Respiratory tract infection	27 (67.5)	3 (33.3)	2.317	0.128
Bloodstream infection	6 (15.0)	5 (55.6)	4.807	0.028
Urinary tract infection	3 (7.5)	0 (0.0)	0.006	0.937
Infection at other sites	4 (10.0)	1 (11.1)	<0.001	1.00
Pre-infection related treatment				
Mechanical ventilation	22 (55.0)	3 (33.3)	0.649	0.420
Central venous catheterization	12 (30.0)	5 (55.6)	1.140	0.286
Number of antibiotic agents	4 (2–5.75)	5 (1.5–5.5)	−0.130	0.909
Glucocorticoid therapy	11 (27.5)	5 (55.6)	1.509	0.219
Relevant examination results at the time of infection				
WBC (×10^9^/L)	10.005 (7.38–12.30)	7.56 (5.17–9.59)	−1.988	0.047
Neutrophil count (×10^9^/L)	5.79 (4.17–7.11)	5.24 (2.83–7.85)	−0.090	0.929
Lymphocyte count (×10^9^/L)	3.01 (2.23–3.75)	1.94 (0.995–3.005)	−2.285	0.020
Number of hospitalizations in the previous 3 months	2 (1–3)	5 (3.5–7.5)	−3.513	<0.001

## Data Availability

The original contributions presented in the study are included in the article material, further inquiries can be directed to the corresponding author.
